# Supplementary material for: Microwave-Assisted Solvothermal Synthesis of Mo-Doped TiO2 with Exceptional Textural Properties and Superior Adsorption Kinetics
Source: Nanomaterials (Basel). 2022 Jun 15;12(12):2051. doi: 10.3390/nano12122051 (PMC9227794; doi:10.3390/nano12122051)
Supplement: Supplementary file 1 [file nanomaterials-12-02051-s001.zip › nanomaterials-1759825-supplementary.pdf]

nanomaterials

Supplementary Material

Microwave-Assisted Solvothermal Synthesis of Mo-Doped TiO_2 with Exceptional Textural Properties and Superior Adsorption Kinetics

Yahia H. Ahmad [1], Aymen S. Abu Hatab [1,2], Assem T. Mohamed [1], Mohammed S. Al-Kuwari [3], Amina S. Aljaber [1] and Siham Y. Al-Qaradawi [1,*]

[1] Department of Chemistry and Earth Sciences, College of Arts and Sciences, Qatar University, Doha 2713, Qatar; yahiashoeb@qu.edu.qa (Y.H.A.); aymenabuhatab@hotmail.com (A.S.A.H.); asemtaha92@gmail.com (A.T.M.); a.s.aljaber@qu.edu.qa (A.S.A.)

[2] Department of Chemistry, Faculty of Science, Universiti Putra Malaysia UPM, Serdang 43400, Selangor, Malaysia

[3] Ministry of Municipality and Environment, Doha 7634, Qatar; msakuwari@mm.gov.qa

* Correspondence: siham@qu.edu.qa, Tel.: +974-44034666

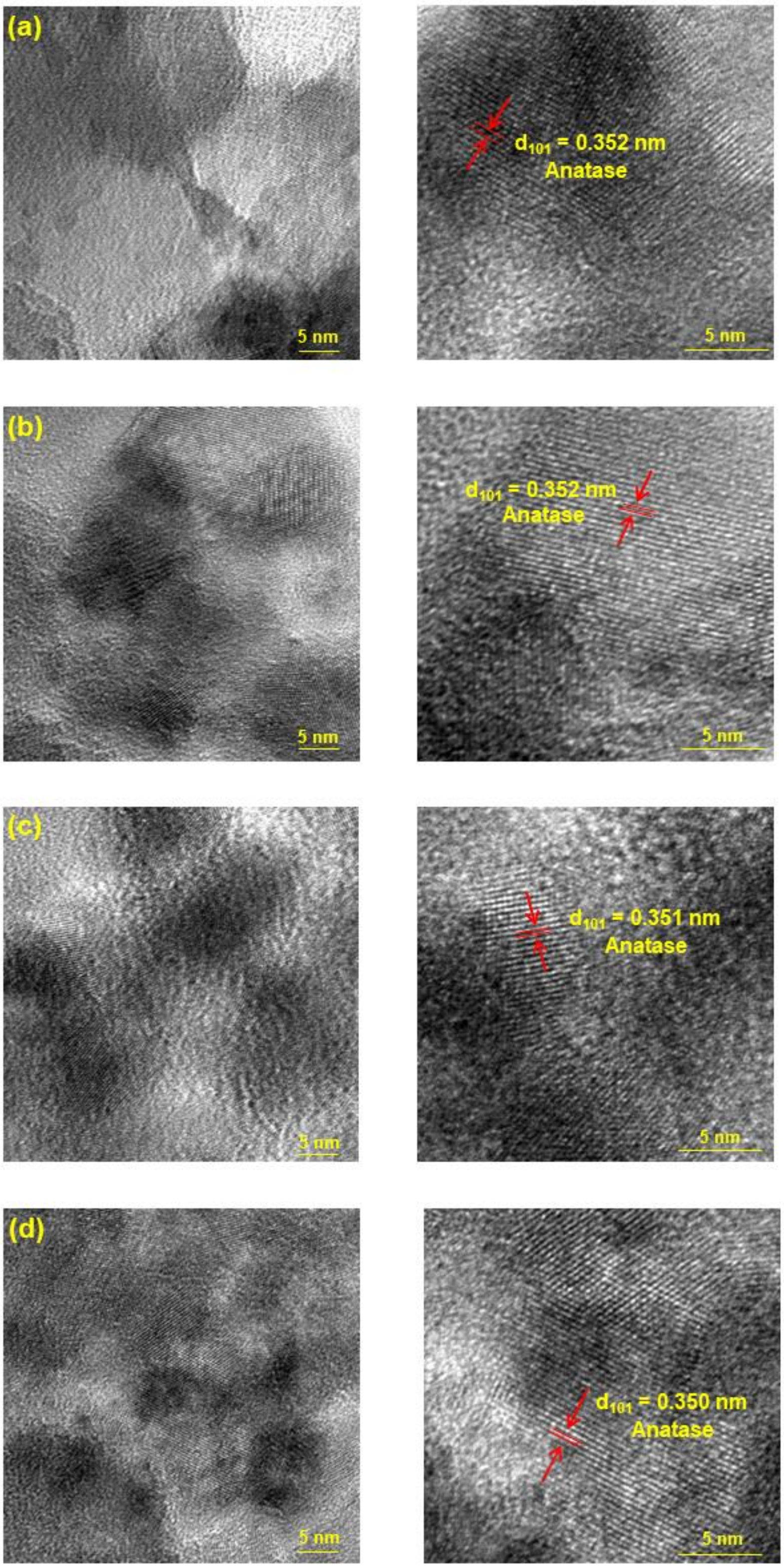

Figure S1. High resolution TEM images of (**a**) TiO_2, (**b**) TiO_2-Mo-0.9, (**c**) TiO_2-Mo-1.5, and (**d**) TiO_2-Mo-2.2.

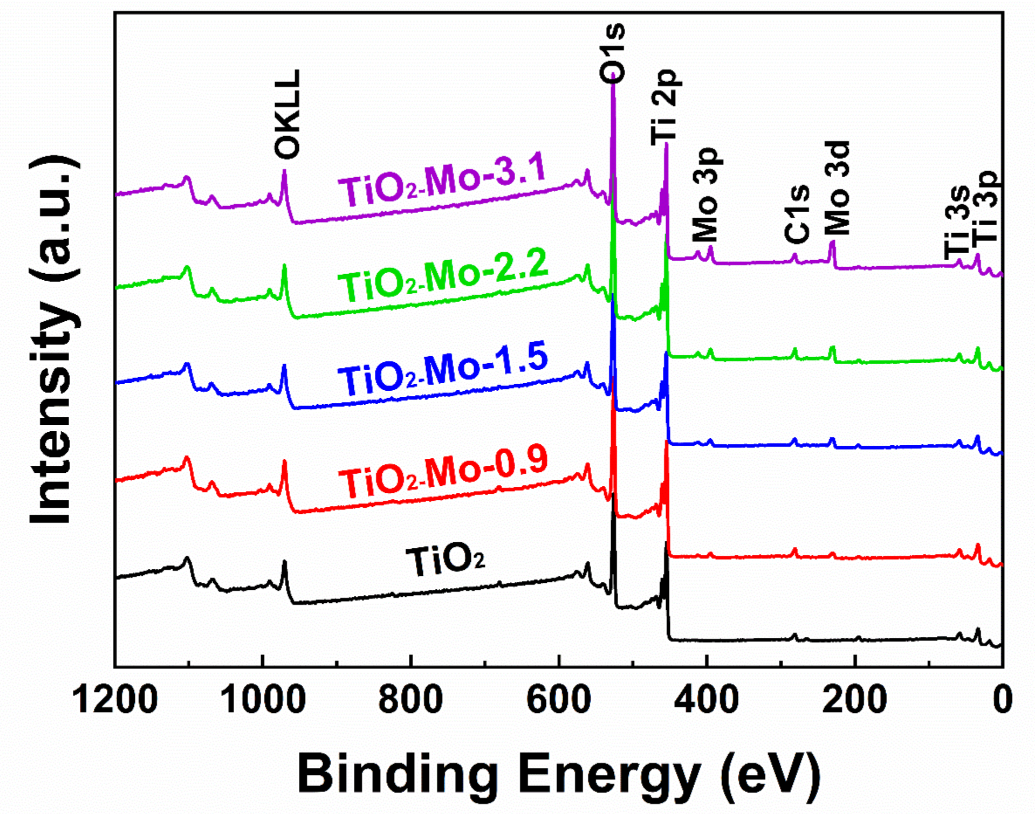

Figure S2. XPS survey spectra of TiO_2 and Mo-TiO_2.

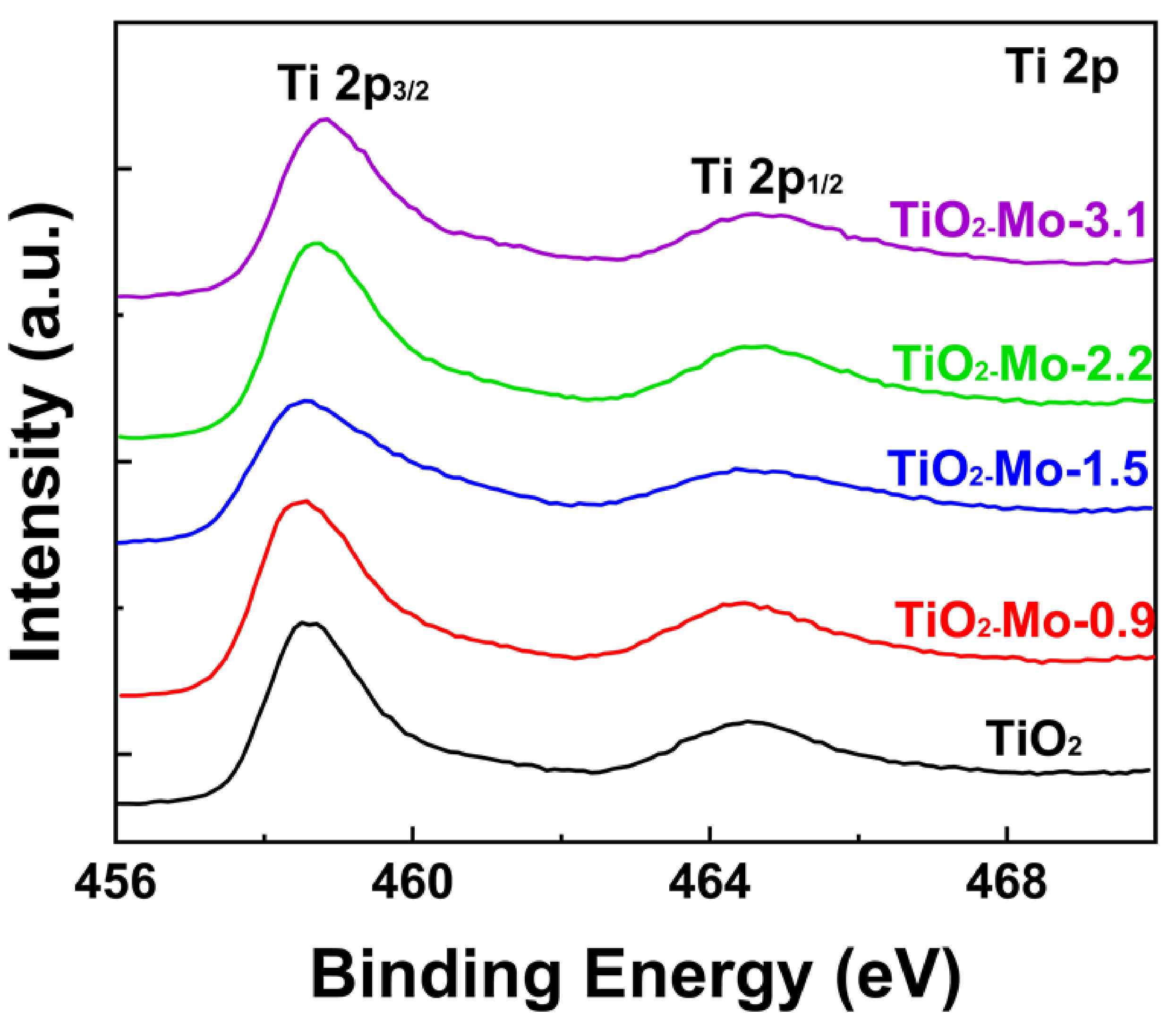

Figure S3. High-resolution XPS spectra of Ti 2p region in all samples.

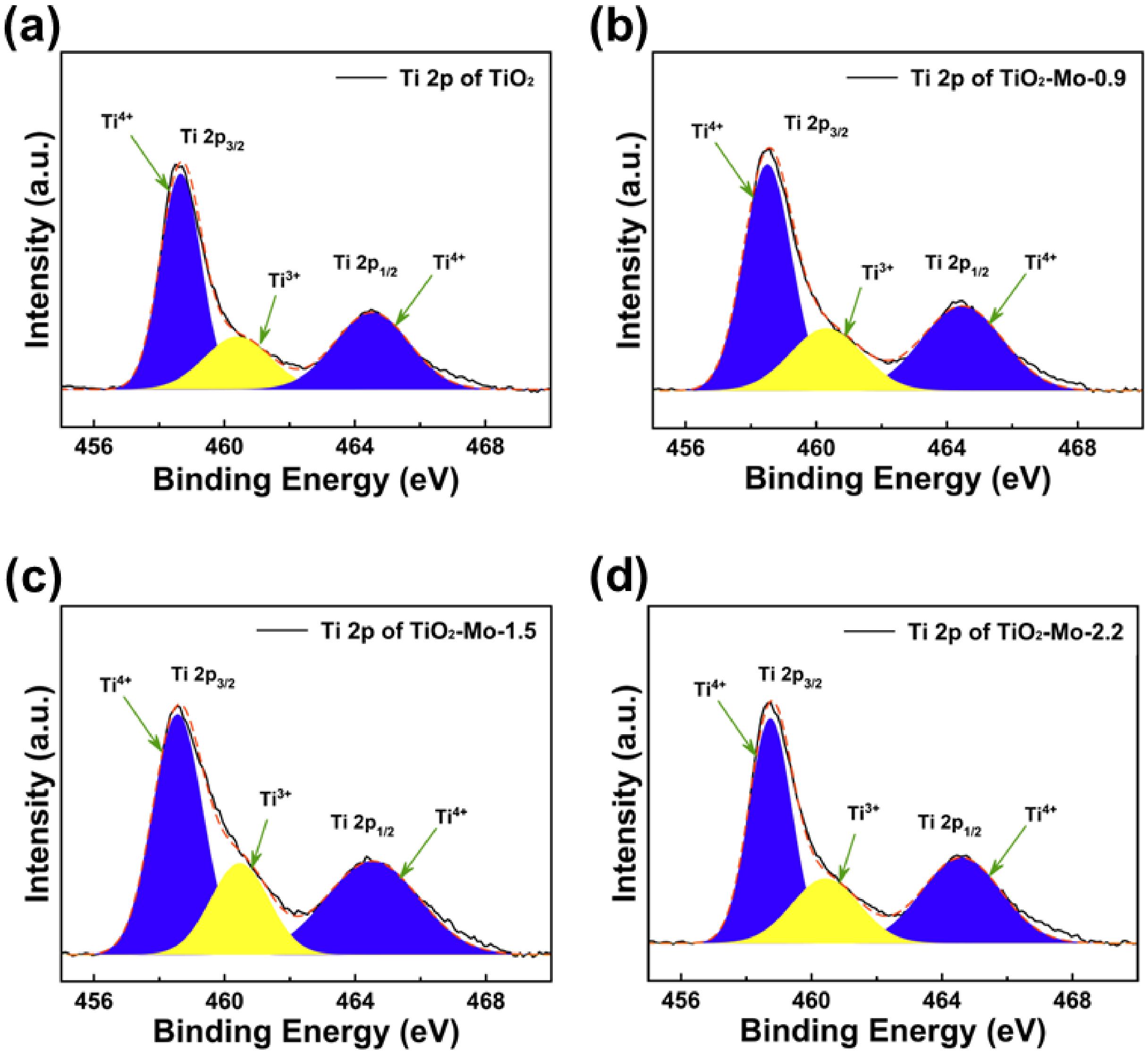

Figure S4. XPS deconvolution of Ti 2p core level in (**a**) TiO_2, (**b**) TiO_2-Mo-0.9, (**c**) TiO_2-Mo-1.5, and (**d**) TiO_2-Mo-2.2.

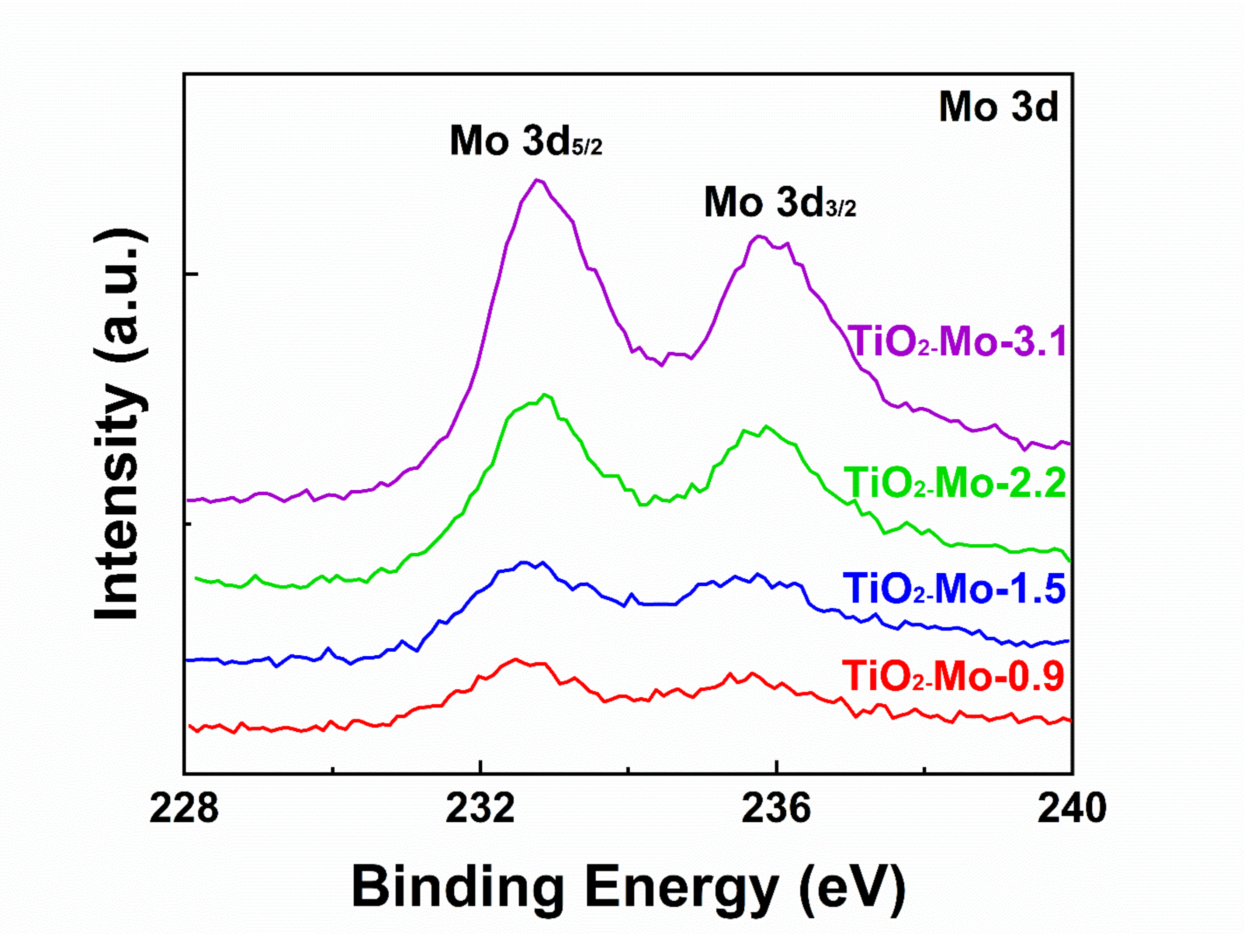

Figure S5. High-resolution XPS spectra of Mo 3d region in studied samples.

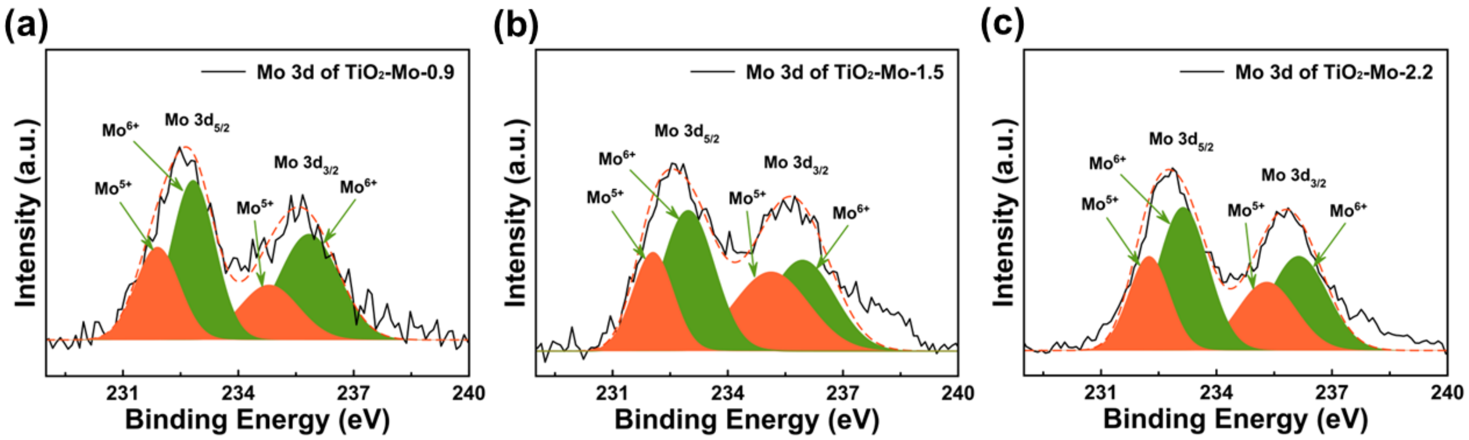

Figure S6. XPS deconvolution of Mo 3d core level in (**a**) TiO_2-Mo-0.9, (**b**) TiO_2-Mo-1.5, and (**c**) TiO_2-Mo-2.2.

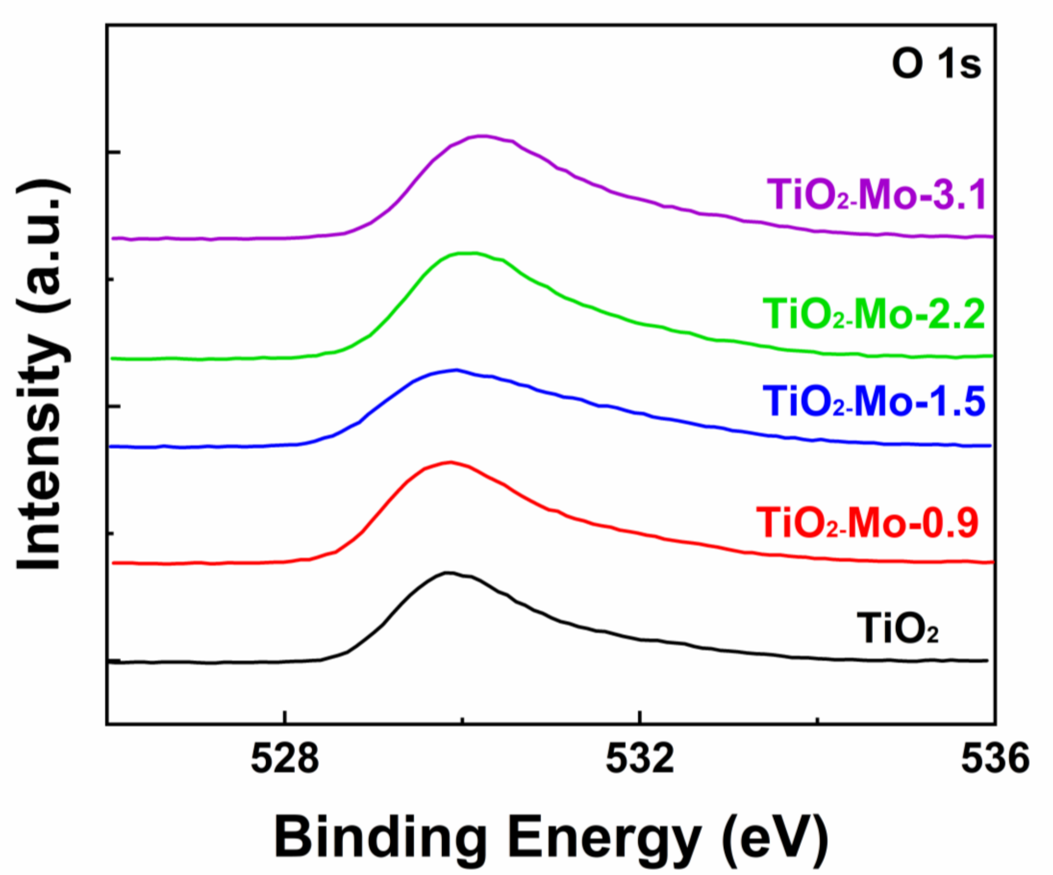

Figure S7. High-resolution XPS spectra of O 1s region in investigated samples.

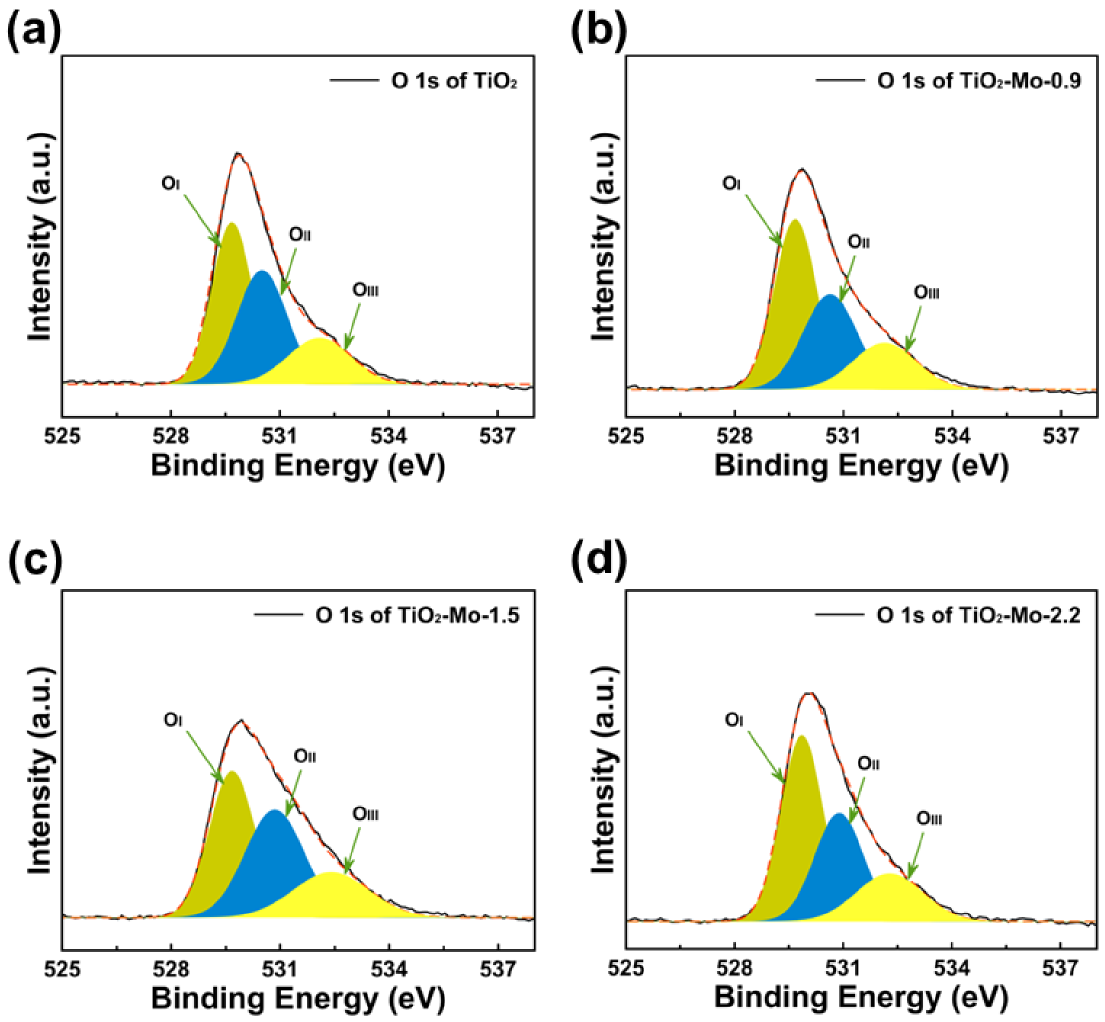

Figure S8. XPS deconvolution of O 1s core level in (**a**) TiO_2, (**b**) TiO_2-Mo-0.9, (**c**) TiO_2-Mo-1.5, and (**d**) TiO_2-Mo-2.2.

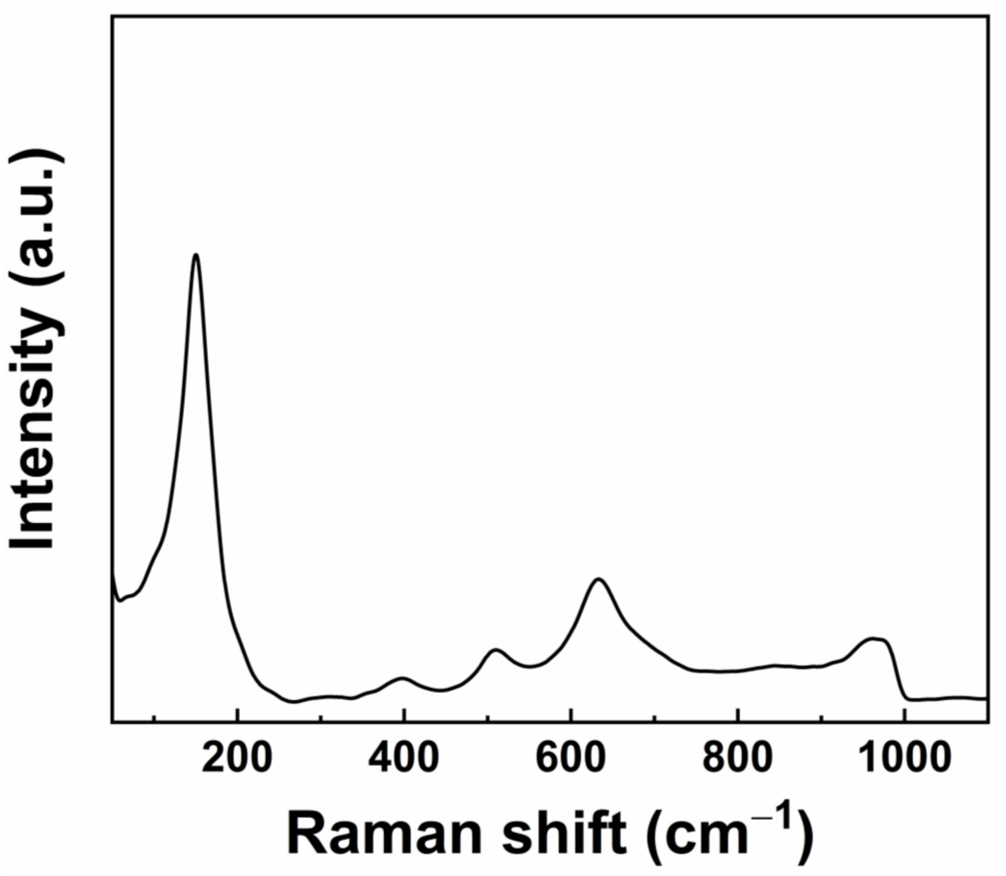

Figure S9. Raman spectrum of TiO_2-Mo-3.9.

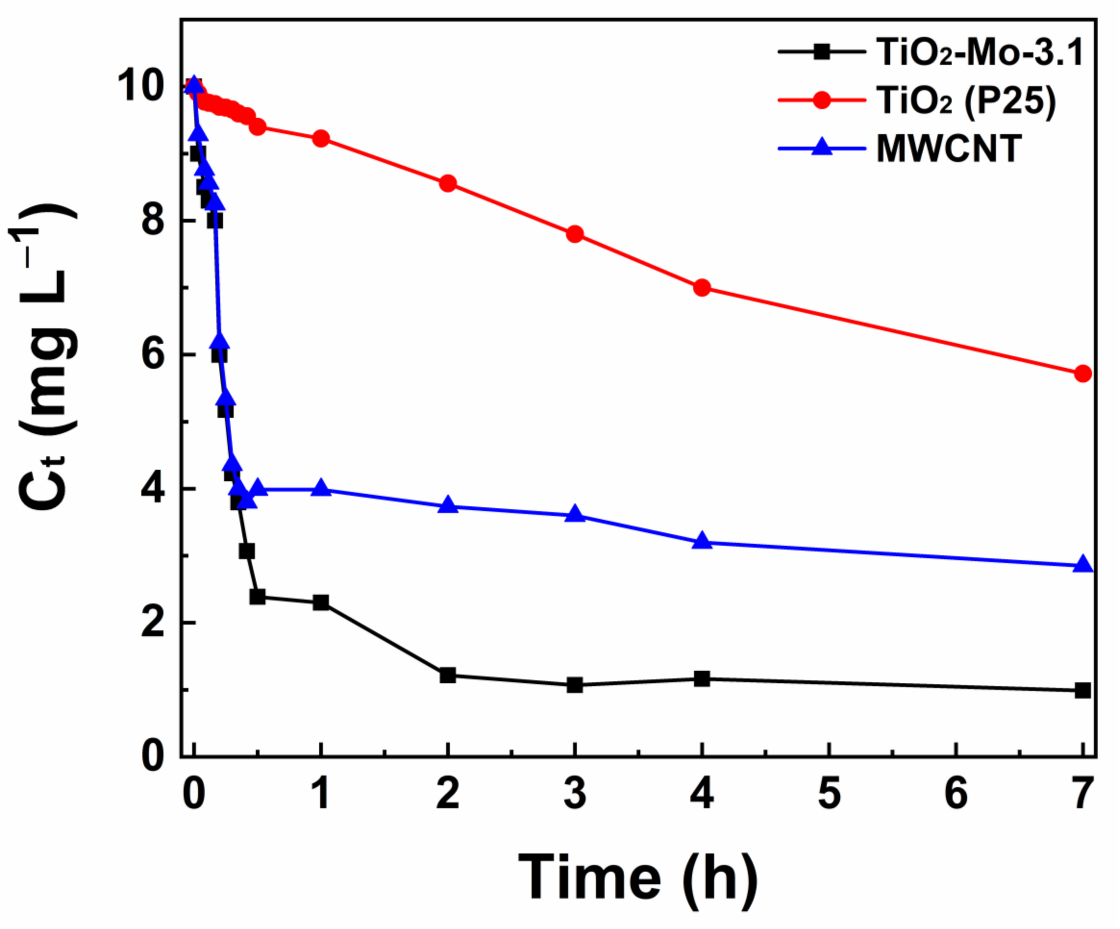

Figure S10. Effect of contact time on the adsorption of Rh.B. for TiO_2-Mo-3.1 compared to commercial adsorbents (C_o = 10 mg L^{-1}, temperature = 298K).
